# Drug distribution along the cochlea is strongly enhanced by low-frequency round window micro vibrations

**DOI:** 10.1080/10717544.2021.1943059

**Published:** 2021-06-26

**Authors:** Samuel M. Flaherty, Ian J. Russell, Andrei N. Lukashkin

**Affiliations:** a Sensory Neuroscience Research Group, School of Pharmacy and Biomolecular Sciences, University of Brighton, Brighton, UK; b Centre for Regenerative Medicine and Devices, University of Brighton, Brighton, UK

**Keywords:** Inner ear drug delivery, cochlea, intratympanic administration, round window membrane, acoustic streaming, assisted diffusion in straight pipes

## Abstract

The cochlea’s inaccessibility and complex nature provide significant challenges to delivering drugs and other agents uniformly, safely and efficiently, along the entire cochlear spiral. Large drug concentration gradients are formed along the cochlea when drugs are administered to the middle ear. This undermines the major goal of attaining therapeutic drug concentration windows along the whole cochlea. Here, utilizing a well-known physiological effect of salicylate, we demonstrate a proof of concept in which drug distribution along the entire cochlea is enhanced by applying round window membrane low-frequency micro vibrations with a probe that only partially covers the round window. We provide evidence of enhanced drug influx into the cochlea and cochlear apical drug distribution without breaching cochlear boundaries. It is further suggested that ossicular functionality is not required for the effective drug distribution we report. The novel method presented here of local drug delivery to the cochlea could be implemented when ossicular functionality is absent or impeded and can be incorporated in clinically approved auditory protheses for patients who suffer with conductive, sensorineural or mixed hearing loss.

## Introduction

**Figure 1.**
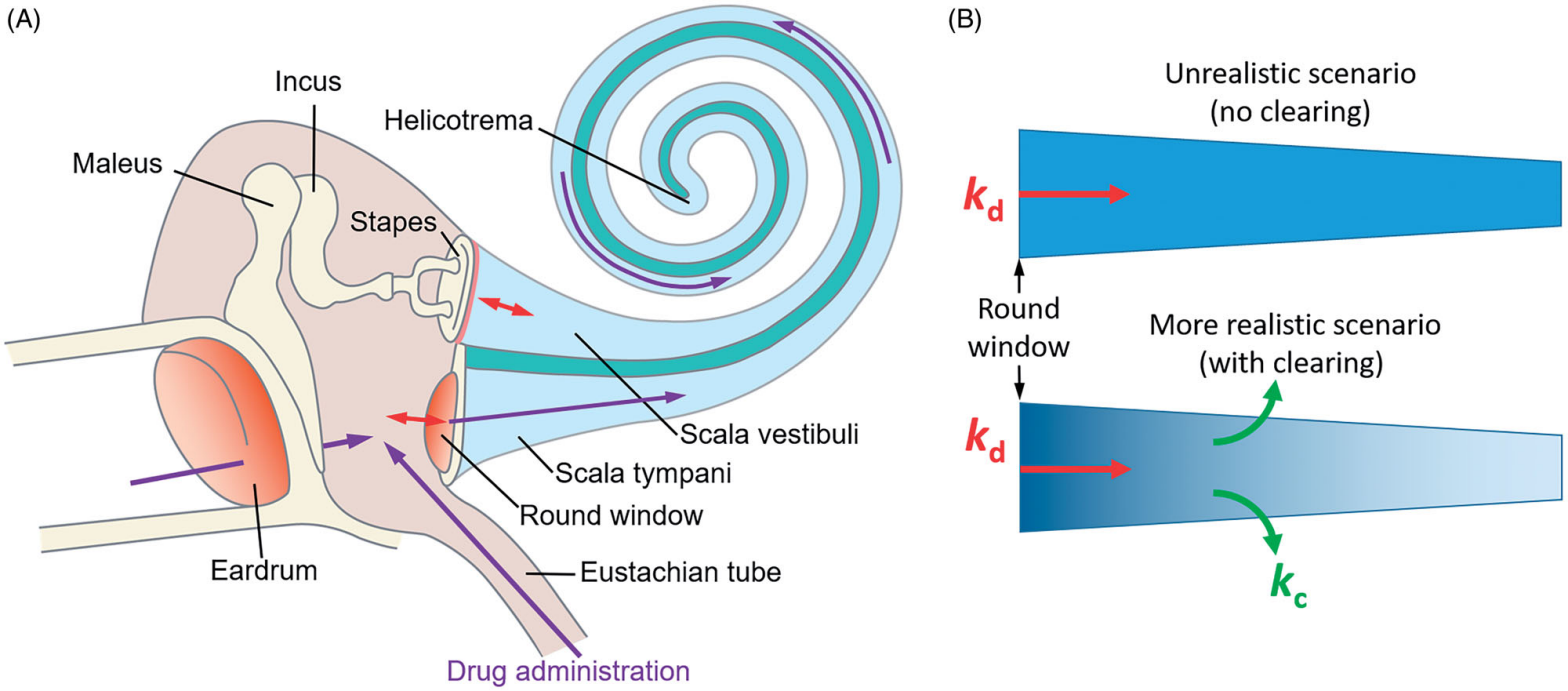
Schematic of the mammalian hearing organ (A) and two scenarios of molecular drug diffusion along the scala tympani (B). (B) Passive molecular diffusion of a drug along the scala tympani is described by a diffusion (kd) and clearing (kc) coefficients. For a given geometry of the scala tympani, the steady-state drug concentration gradient (denoted by the blue color intensity) along it depends only on the ratio kd/kc (Sadreev et al., 2019). (A) is modified from (Lukashkin et al., 2020).

The relative inaccessibility of the human cochlea and its intricate structure requires new drug delivery technologies to be designed to ensure safe, efficient and uniform drug distribution along the entire cochlear spiral (Salt & Plontke, 2009; Rivera et al., 2012; El Kechai et al., 2015; Hao & Li, 2019). The blood-labyrinth barrier hinders the effectiveness of systemic drug administration to the inner ear (Nyberg et al., 2019) and local drug administration becomes increasingly important. Success of the most frequently used topical, intratympanic drug delivery, when drugs are administrated into the middle ear cavity (Figure 1(A)), depends on the ability of the drugs to diffuse into the scala tympani (ST) through the round window membrane (RW) and into the scala vestibuli through the oval window occluded by the stapes (King et al., 2011; Salt et al., 2012; King et al., 2013). If the drug is allowed to diffuse passively along the narrow, extended ST, its concentration, in theory, should become the same within the entire scala after an arbitrary long time (unrealistic scenario, Figure 1(B)) (Sadreev et al., 2019). However, for a drug to be effective, it has to be cleared from the ST into other cochlear compartments (more realistic scenario, Figure 1(B)). Dynamic equilibrium between diffusion and clearing leads to the formation of substantial steady-state, base-to-apex drug concentration gradients along the cochlea (Salt & Ma, 2001; Sadreev et al., 2019), which have been confirmed experimentally for marker ions and contrasting agents (Salt & Ma, 2001; Haghpanahi et al., 2013), corticosteroids (Plontke et al., 2008; Creber et al., 2018) and antibiotics (Mynatt et al., 2006; Plontke et al., 2007). Thus, intratympanic drug administration faces the fundamental problem of limited passive diffusion within the cochlea, which undermines drug efficiency due to the inability of drugs to reach their targets within the therapeutic concentration window.

A few relatively noninvasive techniques for assisting substance mixing along the cochlea have been suggested recently that utilize low-frequency pressure stimulation (Lukashkin et al., 2020), stimulation at acoustic frequencies (Park & Moon, 2014; Shokrian et al., 2020) and ultrasound (Liao et al., 2020) which cause reciprocated movement of the stapes and RW. While the later method relies on the formation of ultrasound-induced microbubbles which can act directly on the RW (Liao et al., 2020), the other techniques require the mobility of the ossicular chain. If the ossicular chain is immobile or malformed then these techniques become non-applicable. In this study we demonstrate that, in this case, micro vibrations of the RW alone can facilitate drug distribution along the cochlear spiral.

## Materials and methods

### Animals and surgery

Animal preparation and signal generation and recording have been described elsewhere (Burwood et al., 2017). Briefly, pigmented guinea pigs of similar weight (350–360 g) and both sexes were anesthetized with the neurolept anesthetic technique (0.06 mg/kg body weight atropine sulfate s.c., 30 mg/kg pentobarbitone i.p., 500 µl/kg Hypnorm i.m.). Additional injections of Hypnorm were given every 40 min. Additional doses of pentobarbitone were administered as needed to maintain a non-reflexive state. The heart rate was monitored with a pair of skin electrodes placed on both sides of the thorax. The animals were tracheotomized and artificially respired with a mixture of O_2_/CO_2_, and their core temperature was maintained at 38 °C with a heating blanket and a heated head holder.

All procedures involving animals were performed in accordance with UK Home Office regulations with approval from the University of Brighton Animal Welfare and Ethical Review Body.

### Signal generation and recording

The middle ear cavity of the ear used for the measurements and salicylate application was opened to reveal the RW. Compound action potentials (CAPs) of the auditory nerve in response to pure tone stimulation were measured from the cochlear bony ridge in the proximity of the RW membrane using Teflon-coated silver wire coupled to laboratory designed and built extracellular amplifier (James Hartley). Thresholds of the N1 peak of the CAP at different frequencies, which corresponds to different distances from the cochlear base (Greenwood, 1990), were estimated visually from an oscilloscope screen using 10 ms pure tone stimuli at a repetition rate of 10 Hz. During the procedure, sound pressure was carefully increased using a manual attenuator control to observe a clear N1 peak of the CAP and then decreased to a sound pressure level when the N1 peak disappeared into the noise floor. This sound pressure level was recorded by the experimental computer. The procedure allowed quick measurements of the CAP thresholds for the entire frequency range between 1–30 kHz well within two minutes.

For acoustic stimulation sound was delivered to the tympanic membrane by a closed acoustic system comprising two Bruel and Kjaer 4134 ½” microphones for delivering tones and a single Bruel and Kjaer 4133 ½” microphone for monitoring sound pressure at the tympanum. The microphones were coupled to the ear canal via 1 cm long, 4 mm diameter tubes to a conical speculum, the 1 mm diameter opening of which was placed about 1 mm from the tympanum. The speculum was sealed in the ear canal. The closed sound system was calibrated in situ for frequencies between 1 and 50 kHz. Known sound pressure levels were expressed in dB SPL re 2 × 1 0 ^−5 ^Pa.

All acoustic stimuli in this work were shaped with raised cosines of 0.5 ms duration at the beginning and at the end of stimulation. White noise for acoustical calibration and tone sequences for auditory stimulation were synthesized by a Data Translation 3010 board (Measurement Computing Corporation, MA) at 250 kHz and delivered to the microphones through low-pass filters (100 kHz cutoff frequency). Signals from the acoustic measuring amplifier (James Hartley) were digitized at 250 kHz using the same board and averaged in the time domain. Experimental control, data acquisition and data analysis were performed using a PC with custom programmes written in MATLAB (MathWorks, MA).

### Salicylate application

5 µl of 100 mM sodium salicylate solution in Hanks’ Balanced Salt Solution were placed on the RW using pipettes. The solution was removed from the RW using paper wicks to observe the wash out effect.

### Round window stimulation

**Figure 2.**
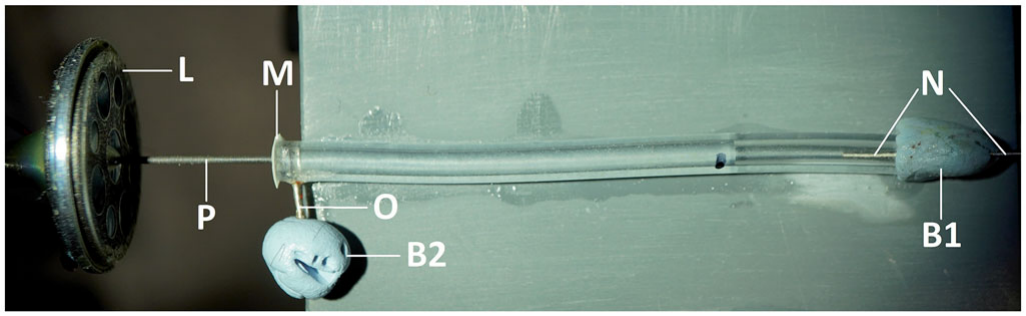
Experimental arrangement for dye diffusion experiments. L – miniature loudspeaker; P – carbon probe; M – latex membrane; O – filling outlet; B1 and B2 – Blu Tack plugs; N – pressure relief needle. See Methods for further details.

A miniature loudspeaker (L, Figure 2) K16-50 Ohm (Visaton GmbH, Haan, Germany) was used to vibrate the RW. The dust cover of the loudspeaker was removed and a probe (P, Figure 2) made of a carbon rod (∼13 mm in length and 0.5 mm in diameter) was glued centrally on the loudspeaker membrane. The probe was perpendicular to the loudspeaker face and remained in this position during experiments to ensure no sideward movements. The probe tip was rounded using a thin layer of superglue preventing RW damage and carbon rod fragmentation. The loudspeaker was fixed to a steel rod, using araldite, and the rod was held in a micromanipulator for a precise probe placement. A programmable synthesiser/signal generator (Philips PM5193) was used to drive the loudspeaker in the experiments. The probe movements versus voltage applied to the loudspeaker were calibrated prior to experiment by focusing a laser vibrometer (CLV-2534, Polytec GmbH, Waldbronn, Germany) at the probe tip along the probe axis and measuring dependence of the probe vibration velocity on the voltage applied to the loudspeaker at the RW stimulation frequencies. The output voltage from the vibrometer was low-pass filtered at 100 kHz, with a sensitivity of 2 mm/s/V. The probe vibration amplitude was calculated by integrating its velocity.

During experiments, the carbon probe was placed at about 45-degrees to the RW because of limited access to the RW. Probe vibrations started immediately after placing salicylate solution on the RW. In the first 20 min period, acoustic CAP threshold recordings were taken every 3–5 min to record the fast action of salicylate at the basal region of the cochlea. Due to the very low frequencies used to vibrate the RW, there was no CAP generated in response to the probe vibrations, allowing recordings of the CAP due to acoustic stimulation to be taken during the RW micro vibrations. After 20 min, the CAP threshold recordings were made every 10 min until a total of 60 min of RW micro vibrations. To washout, the carbon probe was removed, the salicylate was removed from the RW using fine paper wicks and the recovery of CAP threshold to acoustic stimulation was recorded.

### Recording of stapes vibrations

Stapes vibrations were recorded using a laser vibrometer (CLV-2534, Polytec GmbH, Waldbronn, Germany). The laser beam was focused on the stapes head. The output voltage from the vibrometer was low-pass filtered at 100 kHz, with a sensitivity of 2 mm/s/V.

### Fluorescent dye experiments

Lucifer yellow CH, lithium salt (Thermo Fisher Scientific) was used to visualize diffusion in straight water filled pipes (Figure 2). The pipes with an approximate length of 40 mm were constructed using Tygon™ LMT-55 tubing (1.14 mm ID, 0.80 mm wall, Fisher Scientific). An outlet (O, Figure 2) was made with a 25 G needle and inserted through the pipe’s wall close to one end and fixed in place with superglue. A membrane (M, Figure 2), cut from a laboratory latex glove (typical thickness of 0.1 mm), was glued with superglue at the same pipe end making sure that the glue does not cover the open surface of the membrane. The other pipe end was closed with a Blu Tack (Blue-tack.co.uk) plug (B1, Figure 2) to prevent water evaporation and a 25 G needle (N, Figure 2) was inserted through the plug into the pipe to provide pressure relief. The outlet was used to fill the pipe with deionized water to a distance of about 30 mm from the latex membrane and to inject 0.2 µl of 5% Lucifer yellow water solution into the pipe using a pipette. The outlet was closed with a Blu Tack (Blue-tack.co.uk) plug (B2, Figure 2) after the Lucifer yellow injection. Lucifer yellow fluorescence was excited using a 470 nm laser source (Dragon Lasers, Changchun Jilin, China) and still images were taken (Sony α6100 camera, Sony Macro E 30 mm F/3.5 lens) through an optical band pass filter (FB540-10, Thorlabs Inc.) to assess dye diffusion over time. The same miniature loudspeaker K16-50 Ohm (Visaton GmbH, Haan, Germany) with the carbon probe attached as used for the RW stimulation was employed to vibrate the latex membrane in assisted diffusion experiments. The carbon probe touching the membrane was pushed slightly toward inside of the pipes at rest to ensure membrane tension and its relaxation during backward phase of probe strokes. Fluorescence intensity profiles were measured along the pipe axis using Fiji open source image processing package.

## Results

### Low-frequency round window membrane micro vibrations do not elevate hearing thresholds in guinea pigs

**Figure 3.**
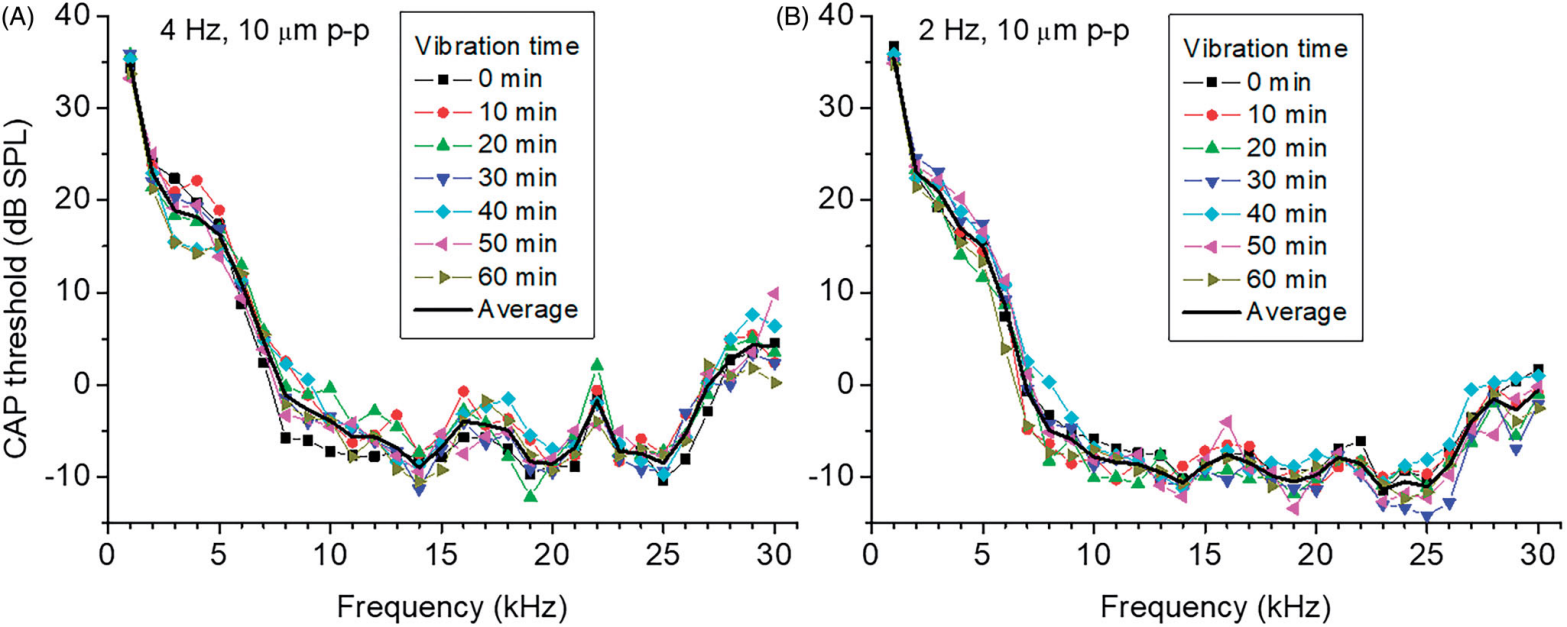
The effect of continuous RW probe vibrations at frequencies of 4 (A) and 2 (B) Hz on acoustic CAP thresholds without application of salicylate solution as a function of acoustic stimulus frequency. Frequency and amplitude of RW probe vibrations are indicated for each panel. Corresponding duration of vibrations is indicated by curves with different symbols. Each curve represents averaged data for 4 preparations (mean value, SD is not indicated for clarity). Solid black curves indicate averaged data (mean value) for all times presented at each panel. Vibration time indicated corresponds to the beginning of each individual CAP threshold curve measurements. It took less than two minutes to record the CAP threshold curve for the entire frequency range 1–30 kHz.

RW stimulation with the carbon probe did not evoke any electrical responses that could be detected by the RW electrode, which made it possible to make continuous CAP threshold measurements to acoustic stimulation throughout the probe vibrations. Hearing sensitivity, assessed by measured CAP thresholds, did not change when 10 μm peak-to-peak continuous probe vibrations were applied to the RW at 2 or 4 Hz for up to 60 min (Figure 3). In our experiments, the probe covered only a small part of the RW. Under these conditions, most of the pressure relief during the probe movement is through the RW area not occluded by the probe. The generated far-field pressure component is small and cochlear excitation is due mainly due to RW near-field pressure, which excites a conventional traveling wave at acoustic frequencies (Weddell et al., 2014). Had a significant far-field pressure been generated, it would cause a stapes movement. However, we were not able to detect any stapes responses above the measurement noise floor of ∼0.1 nm during the RW probe vibrations either at 2 or 4 Hz. As indicated by measurements from the RW electrode, the near-field pressure did not excite the cochlear sensory apparatus at the frequencies of 2 and 4 Hz used in our experiments, even for relatively large 10 µm RW probe displacement. A consequence of this finding is that even large probe induced vibrations of the RW membrane at these frequencies should be safe and unlikely to produce hearing loss (see Discussion for detailed analysis).

### Round window membrane micro vibrations promote drug distribution along the cochlear spiral

The ability of micro vibrations of the RW to improve drug distribution along the cochlear spiral was demonstrated in our experiments with the application of salicylate to the RW. Salicylate readily diffuses through the RW (Borkholder et al., 2014; Sadreev et al., 2019). To monitor salicylate diffusion along an intact guinea pig cochlea *in vivo*, we utilized the suppressive effect of salicylate on cochlear amplification by blocking the outer hair cell (OHC) somatic motility (Russell & Schauz, 1995; Hallworth, 1997). Salicylate competitively binds the motor protein prestin, essential for OHC motility, by repelling Cl^-^-ions and preventing interaction with the anion-binding site (Oliver et al., 2001). We measured the elevation of CAP thresholds caused by salicylate at different frequencies of acoustic stimulation, which, due to cochlear tonotopicity, corresponds to different distances from the RW (Greenwood, 1990). Thus, through measuring the CAP threshold elevations we could assess the spread of salicylate along the cochlea when it was applied to the RW.

**Figure 4.**
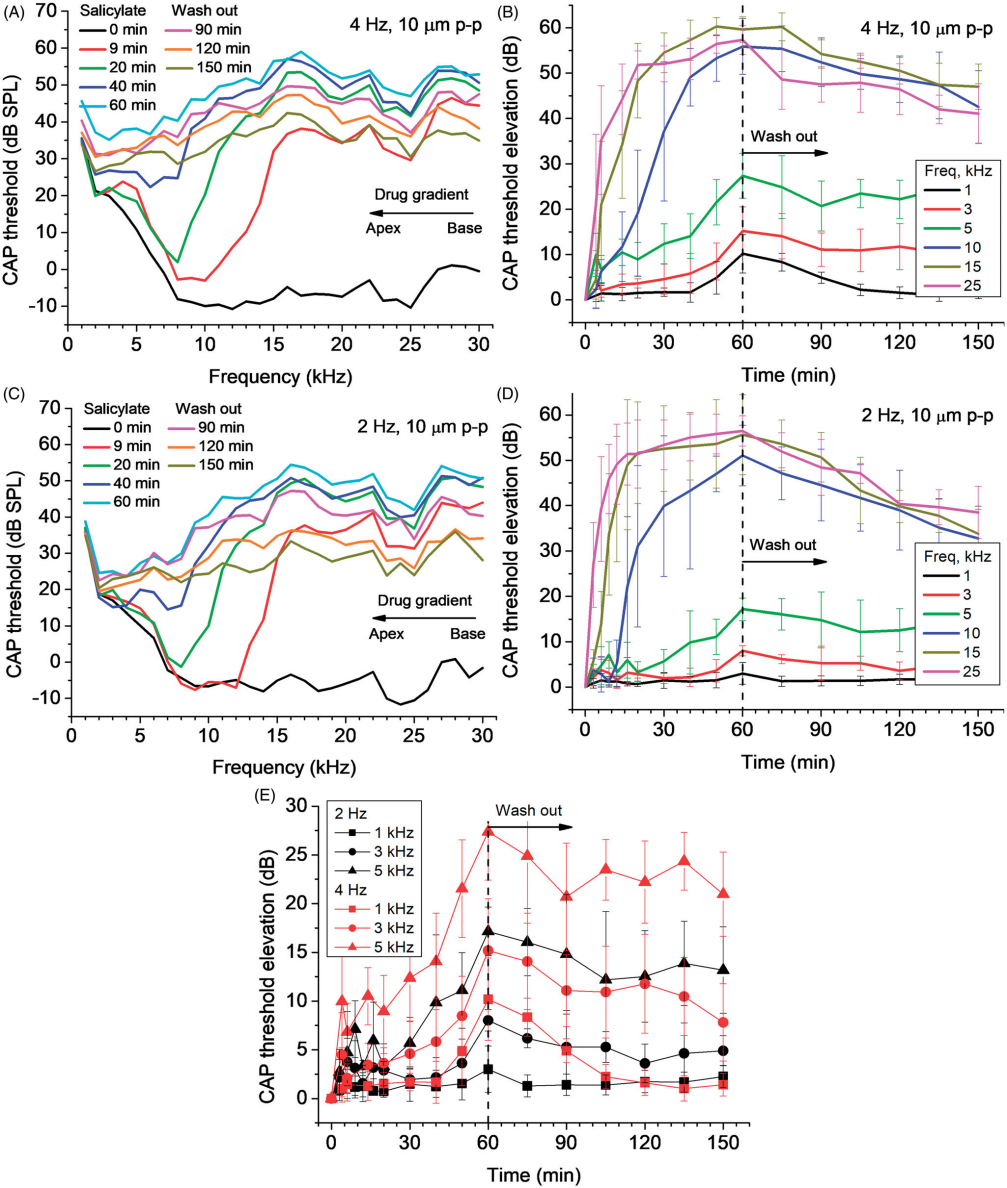
Effect of combined application of 5 µl, 100 mM salicylate solution and continuous RW probe vibrations at frequencies of 4 Hz (A, B, E) and 2 Hz (C, D, E). Salicylate was applied at time zero and RW probe vibrations started at the same time. Salicylate was washed out after 60 min. (A, C). CAP thresholds for different times of salicylate application/RW vibrations (color coded curves) as a function of acoustic stimulus frequency (mean values, SDs are not shown for clarity, *N* = 4). (B, D). CAP threshold elevations relative to the thresholds before salicylate application (time zero) for a few acoustic stimulus frequencies (colour coded curves) which correspond to different locations along the cochlea (mean ± SD, *N* = 6 and 4 for (B) and (D) respectively). (E). CAP threshold elevations relative to the thresholds before salicylate application (time zero) for acoustic stimulus frequencies (different symbols) corresponding to apical half of the cochlea (mean ± SD, *N* = 6 and 4 for 4 and 2 Hz of probe vibrations respectively). Statistically significant (*p* < .05, unpaired *t*-test) differences between the threshold elevations for probe vibrations at 4 and 2 Hz are observed after 60 min of salicylate application/RW vibrations.

When 5 µl of 100 mM salicylate solution was applied to the RW (Figure 4), it caused a rapid increase followed by saturation of CAP thresholds for high frequency tones with the characteristic frequency place situated below or close to the RW (e.g. 25 kHz, Figure 4(B,D)). Over time, CAP threshold elevation gradually spreads to lower frequencies (Figure 4(A,C)) indicating salicylate diffusion into the cochlear apex. Salicylate did not cause elevation of the CAP threshold responses for frequencies below 5 kHz, which corresponds to about 45% of the total cochlear length from the base, when it diffused through the cochlea passively (Sadreev et al., 2019). The calculated gradient of base-to-apex salicylate concentration was about 13 orders of magnitude. When, however, placement of salicylate solution on the RW was followed by RW probe vibrations at frequencies of 2 and 4 Hz, the CAP threshold was elevated throughout the entire 1–30 kHz frequency range tested (Figure 4). The CAP threshold elevation did not saturate and was still rising for frequencies below 5 kHz (Figure 4(B,D)) indicating continuous increase in salicylate concentration in this cochlear region even after 60 min of the probe vibration. This corresponds to about 25% of the total cochlear length from the apex (Greenwood, 1990). Partial recovery of the CAP thresholds during washing out salicylate from the RW after 60 min of its application (Figure 4) provided confirmation that the integrity of the sensory cells was preserved and the CAP threshold elevation after joint salicylate application and RW probe vibrations was not caused by the later.

### Drug distribution along the cochlea length depends on the frequency of round window micro vibration

During combined application of salicylate solution to the RW and RW probe vibrations, the CAP threshold elevations increase when the frequency of the RW probe vibrations is increased (Figure 4). This is particularly evident for the lowest frequencies of acoustic stimulation (Figure 4(E)). For the same acoustic frequencies (i.e. cochlear locations), the total CAP threshold elevations after 60 min of combined salicylate application and RW probe vibrations at 4 Hz were significantly higher (*p* < .05, unpaired *t*-test) than the threshold elevations observed during probe vibrations at 2 Hz. This frequency dependence confirms that increase in the CAP thresholds at frequencies which correspond to more apical cochlear locations and, hence, enhanced diffusion of salicylate to the cochlear apex, was not due to placement of the probe alone and probe vibrations were required to observe the effect.

### Comparison between different techniques of drug delivery through the round window membrane

**Figure 5.**
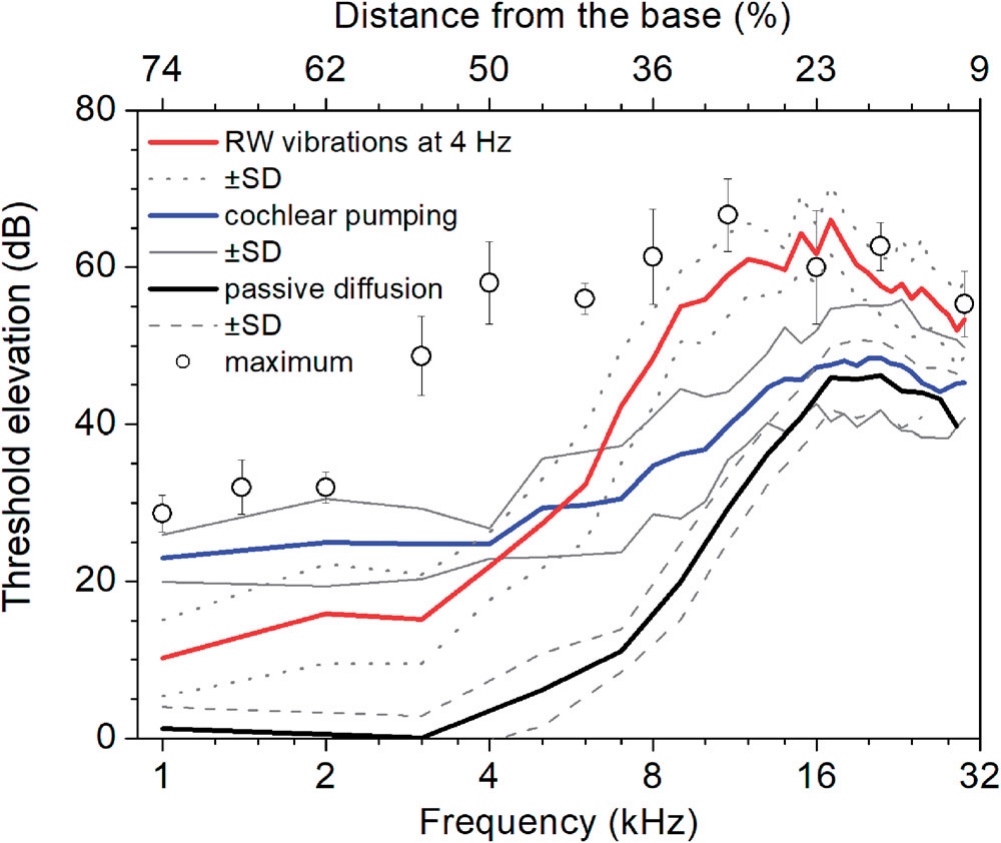
Comparison between different techniques of drug delivery through the RW. Frequency dependence of the CAP threshold elevation after 60 min of 5 µl, 100 mM salicylate solution application for three models of drug distribution: for passive diffusion (black line, mean ± SD, *N* = 5, Lukashkin et al., 2020), during cochlear pumping through application of low-frequency pressure oscillations to the ear canal (35 min of the total pumping time) (blue line, mean ± SD, *N* = 5, from Lukashkin et al., 2020) and continuous RW probe vibrations at 4 Hz (red line, mean ± SD, *N* = 6) are shown. Open circles show maximal increase of the CAP thresholds after complete block of the cochlear amplifier by application of 5 µl of 1 M salicylate solution to the RW (mean ± SD, *N* = 3) (Sadreev et al., 2019).

When 5 µl of 100 mM salicylate solution was applied to the RW and salicylate is allowed to diffuse passively along the cochlear, it does not cause the CAP threshold elevations for frequencies of acoustic stimulation below 5 kHz (Figure 5, black line; Sadreev et al., 2019). This effectively means that salicylate is not able to reach the apical 50% of the cochlea at any effective concentrations. Combined application of salicylate and low-frequency (4 Hz) pressure oscillations to the ear canal (cochlear pumping), which causes large amplitude (80 µm peak-to-peak), movement of the stapes and reciprocal movement of the RW, causes elevation of CAP thresholds within the entire frequency range tested (Figure 5, blue line; Lukashkin et al., 2020). This indicates the ability of the cochlear pumping to distribute salicylate evenly along the entire cochlea. Joint application of salicylate and RW probe vibrations (4 Hz, 10 µm peak-to-peak amplitude) reported in this study causes CAP threshold elevations for the frequencies corresponding to the cochlear apex (Figure 5, red line) indicating enhanced drug diffusion during the RW vibration. These threshold elevations at the cochlear apex (below 5 kHz) were smaller than those observed during cochlear pumping, probably because of the smaller RW probe vibration amplitude (10 µm peak-to-peak) compared to the stapes vibration amplitude (80 µm peak-to-peak). However, the CAP threshold elevations for the basal half of cochlea (frequency of acoustic stimulation above 5 kHz) observed during the RW probe vibrations exceed those recorded during both passive salicylate diffusion and cochlear pumping. These high-frequency thresholds elevations during the RW vibrations are, in fact, close to the maximum threshold elevations after complete block of the cochlear amplifier by application of 1 M/l salicylate solution (Figure 5, circles; Sadreev et al., 2019) and indicate higher basal concentrations, i.e. influx of salicylate into the ST. Therefore, the RW probe vibrations not only promote drug diffusion into the cochlea apex but also enhance salicylate passage through the RW.

### Passive and assisted diffusion of fluorescent dye

**Figure 6.**
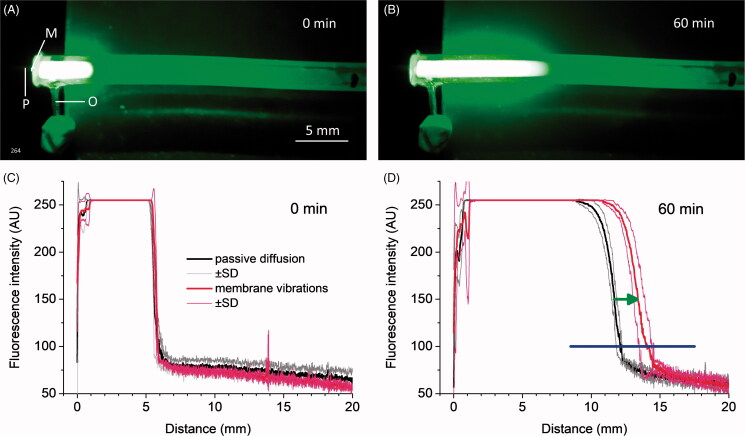
Distribution of Lucifer yellow in a straight pipe during passive diffusion and during vibrations of a membrane at a pipe end. (A). Fluorescence immediately after injections of 0.2 µl of 5% Lucifer yellow water solution into the pipe through outlet O. M – latex membrane; P – carbon probe. (B). Fluorescence after 60 min of membrane vibrations at 10 Hz with 40 µm peak-to-peak carbon probe movements. (C). Overlapping fluorescence intensity profiles measured along the pipe axis for all passive diffusion (black line, mean ± SD, *N* = 3) and membrane vibration (red line, mean ± SD, *N* = 3) experiments indicating the same initial conditions immediately after the dye injection. (D). Fluorescence intensity profiles after 60 min of passive dye diffusion (black line, mean ± SD, *N* = 3) and membrane vibrations (red line, mean ± SD, *N* = 3). The same experiments as in C. Green arrow indicates additional spread of the diffusion front during membrane vibrations. Blue horizontal bar indicates the spread of statistically significant increase (unpaired *t*-test for 0.5 mm bins, *p* < .05) in the fluorescence intensity/dye concentration which is observed during membrane vibrations.

To gain insight into the mechanism of enhanced drug diffusion in our experiments with the RW vibrations, we compare the speed of passive, molecular diffusion of Lucifer yellow along water filled straight pipes and its diffusion assisted by micro vibrations of a membrane covering one end of the pipes (Figure 6(A,B)). The pipes were water filled to ∼30 mm from the membrane and their internal cross-sectional area was ∼1.02 mm^2^ which correspond to the length and average cross-sectional area of the human ST, respectively (Thorne et al., 1999). Fluorescence intensity profiles, obtained immediately after injection of 0.2 µl of the dye, were closely similar in all experiments (Figure 6(C)), indicating the initial conditions remained constant for all sets of measurements. Small, 40 µm peak-to-peak vibration of the membrane at 10 Hz over 60 min enhanced dye distribution compared to passive diffusion (Figure 6(D)). The additional spread of the diffusion front was small (about 1.7 mm for fluorescence intensity of 150 AU, green arrow in Figure 6(D)). However, due to nonlinear dispersion of the diffusion front, this small additional spread led to a statistically significant increase (unpaired *t*-test for 0.5 mm bins, *p* < .05) in the fluorescence intensity, i.e. dye concentration, over a much wider range of 9 mm (blue horizontal bar, Figure 6(D)).

## Discussion

This is a proof-of-concept report which demonstrates that vibrating the partially occluded RW at low frequencies of 2 and 4 Hz and with an amplitude of 5 µm facilitates drug distribution along the cochlear spiral. Finding optimal and safe parameters for the RW vibrations was outside the study’s scope. However, we can conclude that, for the range of stimulation parameters used, and within the timeframe of experiments, drug diffusion was enhanced with increasing RW stimulus frequency without affecting the neural thresholds. This frequency dependence of drug distribution also indicates that placement of the RW probe did not rupture the RW. At the same time, the enhanced effect on the CAP threshold elevation in the basal half of the cochlea, produced by the RW vibrations (Figure 5), compared to passive diffusion (Sadreev et al., 2019) and cochlear pumping (Lukashkin et al., 2020), suggests that the RW drug permeability for salicylate was increased during direct mechanical stimulation of the RW.

RW vibration stimulation alone did not elicit electrical responses which could be recorded at the RW, including CAPs associated with afferent fibre/inner hair cell excitation or cochlear microphonic potentials dominated by basal turn OHC mechanoelectrical transducer currents (Patuzzi et al., 1989; Cheatham et al., 2011). Previously, very large cochlear microphonic potentials in response to 5 Hz *acoustic* stimulation were recorded from the cochlear apex but not from the cochlear base in guinea pigs suggesting excitation of the OHCs in this tonotopic frequency place (Salt et al., 2013). We argue, however, that excitation of sensory hair cells due to the low-frequency RW vibrations was minimal in our experimental configuration. The RW probe diameter (0.5 mm) and its cross-sectional area (0.2 mm^2^) were much smaller than the dimensions and area of the RW in guinea pigs (Ghiz et al., 2001; Wysocki & Sharifi, 2005), and the probe covered only a small part of the RW. Under these conditions, most of the pressure relief during the probe movement was through the RW area not occluded by the probe (Weddell et al., 2014) and the average alternating far-field pressure, $P_{M},$ generated within the cochlea was small. This was confirmed by the absence of stapes responses, which were below the measurement noise floor (∼0.1 nm) during the probe vibrations at the RW either at 2 or 4 Hz. The magnitude of $P_{M}$ will depend on the stiffness, S, of the freely moving area, A, of the RW and its volume velocity, q, as (Weddell et al., 2014)

(1) $$P_{M}=\frac{Sq}{i\omega A^{2}}.$$

Even if a small far-field pressure was generated in our experiments due to finite stiffness, S, of the RW, which did not generate measurable stapes vibrations, then it still would not lead to a significant excitation of the BM. The frequencies of 2 and 4 Hz used in our experiments are notably below the helicotrema cutoff frequency in guinea pigs (Marquardt et al., 2007) and will be filtered out by the helicotrema, preventing BM excitation at these frequencies and damage to the cochlear sensory cells during the RW probe stimulation. The role of the cochlear aqueduct in pressure relief at frequencies 2−4 Hz, used to vibrate the RW in our experiments, should be small because the time constant for inner ear pressure relaxation due to the cochlear aqueduct is in the order of seconds (Thalen et al., 2002; Feijen et al., 2004). The potential influence of mass and viscosity of a relatively large volume of fluid placed on the RW in our experiments should also be small. Dai et al. (2008) demonstrated that the cochlear impedance is still stiffness-dominated at low frequencies when the middle ear is filled with fluid. i.e. when the RW is loaded with fluid. Dai et al. did not make measurements at frequencies as low as the 2−4 Hz used in our experiments but asymptotic behavior of their magnitude transfer functions at low frequencies clearly indicates stiffness dominated responses.

Vibrations of the partially occluded RW at acoustic frequencies excite the basilar membrane with conventional travelling waves. A jet-like, near-field component, $P_{N},$ of a complex pressure field near the RW is the proposed mechanism of stimulation (Weddell et al., 2014).$P_{N}$ is proportional to the fluid density, ρ, and to the acceleration of the probe, iωq, and an indicative overall magnitude of $P_{N}$ can then be defined as

(2) $$P_{N}=\text{iωρq}.$$

Because RW stimulation with the carbon probe did not evoke any cochlear microphonic potentials from the basal OHCs that could be detected by the RW electrode (Patuzzi et al., 1989; Cheatham et al., 2011), we conclude that this near-field component did not excite the basilar membrane at frequencies of 2 and 4 Hz used in our experiments which also resulted in lack of excitation of the cochlear sensory apparatus and absence of any probe induced hearing loss, even for relatively large 10 µm peak-to-peak RW probe displacements. It is worth noting, that the near-field pressure, $P_{N},$ increases with increasing frequency (Equation 2). This can explain the higher efficiency of 4 Hz RW stimulation compared to 2 Hz (Figure 4(E)) if the near-field pressure component is the main factor facilitating enhanced drug diffusion during vibration of a partially occluded RW.

The question is how this short acting jet-like, near-field pressure component can facilitate drug distribution along the entire cochlea, which is an order of magnitude longer than the near-field pressure spread (Weddell et al., 2014). The fluorescent dye experiments (Figure 6), while being different from the RW stimulation experiments in two important aspects, provide an insight into the underlying physical mechanisms. Firstly, the latex membrane stiffness was much larger than the RW stiffness. Pressure relief in this case was through the open pipe end and a large far-field pressure component was generated within the fluid-filled pipe leading to movement of the entire fluid column. Taylor dispersion (Taylor, 1953) of solvents is observed during oscillatory pipe flows which lead to additional spread of solvents compared to molecular diffusion alone (Aris, 1960; Watson, 1983). It has been demonstrated experimentally that for small-stroke fluid oscillatory movements and dimensions of the human cochlea this effect is small (Dasgupta, 2015), which is confirmed by lack of changes in the diffusion front in our experiments (Figure 6(D)). However, when a physical body vibrates in confined spaces, which resembles the geometry of our experiments, the jet-like fluid movement is transformed into a steady fluid streaming which forms vortexes in the vicinity of the vibrating body even at low frequencies (Costalonga et al., 2015). The vortexes can facilitate fluid mixing close to the vibrating body, which is the inner surface of the vibrating membrane in our experiments. Thus, in the fluorescent dye experiments, this mixing should change the boundary condition at the closed pipe end and lead to additional spread of the diffusion front without changing its dispersion (Figure 6(D)). The diffusion front dispersion over the same time is larger for substances with larger diffusion coefficients. Therefore, we can predict that the effective range of increased concentration should be larger for salicylate used in our experiments (salicylate diffusion coefficient is 9.59 × 10^−4^ mm^2^/s (Lide (2002)) and for dexamethasone, the most frequently used drug for intratympanic treatment of hearing disorders (dexamethasone diffusion coefficient calculated from Stokes-Einstein equation which, however, underestimates experimental values, is 6.82 × 10^−4^ mm^2^/s), than we observed for Lucifer yellow (diffusion coefficient is 3.1 × 10^−4^ mm^2^/s (Brink & Ramanan, 1985)).

The second major difference between our *in vivo* and fluorescent dye experiments is in the amount of material available for diffusion. The amount of dye was limited by its initial injection. A relatively large volume of 5 µl of salicylate solution was placed on the outer surface of the RW *in vivo*. Hence, an additional amount of salicylate could enter the ST down its concentration gradient when the salicylate concentration in the immediate vicinity of the inner surface of the RW dropped due to enhanced mixing because of the vortex formation described above and because the RW permeability increased during its mechanical stimulation (e.g. Park & Moon, 2014; Liao et al., 2020). This facilitated additional influx of salicylate could increase its concentration at the cochlear base, which is indicated by higher basal CAP threshold elevations observed in our experiments (Figure 5). The increase in salicylate concentration changes the diffusion boundary condition and promotes diffusion of salicylate to the cochlear apex. It should be noted that salicylate was utilized in this study due to its well documented physiological effects. However, it is a difficult drug to distribute along the cochlea because it is cleared rapidly from the ST (Sadreev et al., 2019). It is anticipated that drugs, which are better retained in the ST, will be redistributed along the cochlea even more quickly and efficiently (Salt & Ma, 2001; Sadreev et al., 2019).

This work is a proof of concept study and it remains to be demonstrated that the RW micro vibrations can promote distribution of substances for cochleae of the human cochlea’s size and for stimulation parameters that are safe for human cochlear function. If this drug delivery method is effective in human patients, it could be used to deliver and distribute drugs along the cochlea when cochlear pumping (Lukashkin et al., 2020) cannot be applied. For example, when the ossicular functionality is absent or impeded, e.g. after injection of high concentrations of hydrogel formulations into the middle ear (e.g. Piu et al., 2011; Schilder et al., 2019). Cochlear drug delivery utilizing micro vibrations of the RW could be particularly useful in patients with round window vibroplasty (e.g. Beltrame et al., 2014) if a part of the RW is left available for drug diffusion from the middle ear. In this case a vibrator is already present at the RW and any additional interventions required are minimal.

## Data Availability

Data is available on request through the University of Brighton Research Data Repository at https://researchdata.brighton.ac.uk/
